# Contemporary Pharyngeal and Invasive *emm*1 and Invasive *emm*12 Group A *Streptococcus* Isolates Exhibit Similar *In Vivo* Selection for CovRS Mutants in Mice

**DOI:** 10.1371/journal.pone.0162742

**Published:** 2016-09-09

**Authors:** Wenchao Feng, Mengyao Liu, Daniel G. Chen, Rossana Yiu, Ferric C. Fang, Benfang Lei

**Affiliations:** 1 Department of Microbiology and Immunology, Montana State University, Bozeman, Montana 59718, United States of America; 2 Harborview Medical Center Clinical Microbiology Laboratory and University of Washington School of Medicine, Seattle, Washington, United States of America; University of South Dakota, UNITED STATES

## Abstract

Group A *Streptococcus* (GAS) causes diverse infections ranging from common pharyngitis to rare severe invasive infections. Invasive GAS isolates can have natural mutations in the virulence regulator CovRS, which result in enhanced expression of multiple virulence genes, suppressed the expression of the protease SpeB, and increased virulence. It is believed that CovRS mutations arise during human infections with GAS carrying wild-type CovRS and are not transmissible. CovRS mutants of invasive GAS of the *emm*1 genotype arise readily during experimental infection in mice. It is possible that invasive GAS arises from pharyngeal GAS through rare genetic mutations that confer the capacity of mutated GAS to acquire *covRS* mutations during infection. The objective of this study was to determine whether contemporary pharyngeal *emm*1 GAS isolates have a reduced propensity to acquire CovRS mutations *in vivo* compared with invasive *emm*1 GAS and whether *emm*3, *emm*12, and *emm*28 GAS acquire CovRS mutants in mouse infection. The propensity of invasive and pharyngeal *emm*1 and invasive *emm*3, *emm*12, and *emm*28 SpeB^A+^ isolates to acquire variants with the SpeB^A-^ phenotype was determined during subcutaneous infection of mice. The majority of both invasive and pharyngeal *emm*1 SpeB^A+^ isolates and two of three *emm*12 isolates, but not *emm*3 and *emm*28 isolates, were found to acquire SpeB^A-^ variants during skin infection in mice. All analyzed SpeB^A-^ variants of *emm*1 and *emm*12 GAS from the mouse infection acquired *covRS* mutations and produced more platelet-activating factor acetylhydrolase SsE. Thus, contemporary invasive and pharyngeal *emm*1 GAS isolates and *emm*12 GAS have a similar capacity to acquire *covRS* mutations *in vivo*. The rarity of severe invasive infections caused by GAS does not appear to be attributable to a reduced ability of pharyngeal isolates to acquire CovRS mutations.

## Introduction

Group A *Streptococcus* (GAS) is a major human pathogen that causes both relatively mild pharyngitis and superficial skin infections and potentially lethal, severe invasive infections [1]. Severe GAS infections were frequent and often fatal in the 19^th^ century and reemerged in the 1980s [2]. The reemergence of severe invasive GAS infections in the 1980s is associated with the emergence of a virulent M1T1 clone of *emm*1 GAS [3–5] and virulent *emm*3 GAS [6]. The M1T1 clone of *emm*1 GAS is evolved by the acquisition of DNase Sda1- and superantigen SpeA-encoding prophages and the replacement of a 36-kb chromosomal region of pre-1980 M1 GAS with that of *emm*12 GAS that contains the NADase and streptolysin O genes [7–9]. Contemporary M3 GAS acquired a prophage that encodes the superantigen SpeK and phospholipase A2 SlaA [6]. Since 2000, M89 GAS without the genes for synthesis of the hyaluronic acid capsule has also emerged to cause severe invasive infections in the United Kingdom, as well as the United States, Finland, Iceland, and Portugal [10–12].

Invasive GAS isolates frequently exhibit a greater capacity to invade soft tissues and evade neutrophil responses in association with higher virulence in experimental animal infections in comparison to pharyngeal isolates. Natural acquisition of mutations of the two-component regulatory system CovRS (also known as CrsRS) leads to hypervirulence [13–19]. Invasive GAS isolates frequently carry CovRS mutations [20,21], which appear to arise during human infections with GAS carrying wild-type CovRS and are not transmissible [22]. CovRS negatively regulates many virulence factors, including most of those that are involved in the evasion of innate immunity [23–25]. As results of CovRS mutations, loss of production of the protease SpeB and enhanced production of the hyaluronic acid capsule and platelet-activating factor acetylhydrolase SsE contribute to the phenotype of hypervirulent invasive M1T1 and M3 GAS isolates [26–32].

The emergence of CovRS mutants of invasive M1T1 GAS has been readily demonstrated during experimental mouse infections [8,13,14,17,31–33]. Not all *emm*1 GAS strains are subject to selection of CovRS mutations during skin and soft tissue infections [8]. The DNase *sda*1 and the *hasA* and M protein (*emm*) genes are reported to be required for the selection of CovRS mutants in the M1T1 strain 5448 [31,32]. However, the role of Sda1 in the selection of CovRS mutants of M1T1 GAS has not been confirmed [33]. Among the host factors important for *in vivo* selection of invasive M1T1 GAS CovRS mutants, neutrophils are required for selection of *covRS* mutants in mouse infection, and M1T1 CovRS mutants exhibit greater resistance to neutrophils *in vivo* [17].

Approximately 15 million cases of streptococcal pharyngitis and 10,000 cases of severe invasive GAS disease occur annually in the United States. The basis for the rarity of severe invasive GAS infections compared with common strep throat is not well understood. It is well known that isolates that cause invasive disease are the same as those circulating in the upper respiratory tracts of the community [34], and contemporary pharyngeal *emm*1 isolates from Finland and *emm*1 invasive isolates from diverse geographic areas are closely related to the sequenced M1T1 GAS strain MGAS5005 [9]. However, there are single nucleotide polymorphisms (SNP) and indels among isolates of the M1T1 clone [9,14]. It is possible that invasive GAS arises from rare genetic mutations of pharyngeal GAS that confer the capacity of mutated GAS to acquire *covRS* mutations during infection. MGAS2221, a pharyngeal M1T1 isolate, has been shown to acquire *covRS* mutations during murine infection [14], inconsistent with the possibility. However, this strain was isolated from a patient with scarlet fever and may not represent a typical pharyngitis case. Thus, parallel comparison with multiple invasive and pharyngeal *emm*1 isolates in the capacity to acquire *covRS* mutations should be made to test whether pharyngeal and invasive *emm*1 isolates have differential capacity to acquire *covRS* mutations.

The most common *emm* genotypes associated with contemporary severe invasive infections in the United States are *emm*1, *emm*28, *emm*12, *emm*3, and *emm*11 in 1995–1999 [35], *emm*1, *emm*3, *emm*28, *emm*12, and *emm*89 in 2000–2004 [36], and *emm*1, *emm*12, *emm*28, *emm*89, and *emm*3 in 2005–2012 [37]. CovRS mutations have been frequently detected in clinical isolates of various *emm* genotypes [9,20,21,38]. However, emergence of CovRS mutants in experimental animal infections has been demonstrated only for *emm*1 GAS [8,13,14,17,31–33]. C*ovRS* mutations of an *emm*3 strain are not detected in muscular infection in nonhuman primates; however, GAS isolates tested might be recovered from primates at day 1 after inoculation [39], which may be too soon to accumulate CovRS mutants. Thus, it is not known whether GAS isolates of the other most dominant invasive emm genotypes other than *emm*1 can readily acquire CovRS mutations in experimental animal infection.

This study was designed to determine whether pharyngeal *emm*1 GAS isolates have less propensity to acquire CovRS mutations *in vivo* than invasive *emm*1 GAS and whether *emm*3, *emm*12, and *emm*28 GAS can acquire CovRS mutants in mouse infection. The *emm*89 GAS was excluded for the second question because invasive *emm*89 GAS lacks the hyaluronic acid capsule [10,11] while the capsule has been shown to be critical for *in vivo* selection of *emm*1 CovRS mutants [32]. We first identified isolates that secrete the protease SpeB (SpeB^A+^ for the presence of SpeB activity in culture supernatant) among 176 invasive GAS isolates collected from patients with necrotizing fasciitis (NF) and/or streptococcal toxic shock syndrome (STSS) in 2010–2013 by the CDC *Streptococcus* Laboratory and 50 pharyngitis isolates collected in 2014 by the Harborview Medical Center Clinical Microbiology Laboratory at University of Washington School of Medicine. We then compared the capacity of SpeB^A+^ invasive and pharyngeal *emm*1 isolates to acquire SpeB^A-^ variants during subcutaneous infection in mice. We also examined the capacity of SpeB^A+^ invasive *emm*3, *emm*12, and *emm*28 GAS isolates to acquire SpeB^A-^ variants during mouse infection. We found that the majority of both invasive and pharyngeal *emm*1 SpeB^A+^ isolates and two of three *emm*12 isolates acquired SpeB^A-^ variants during skin infection. Sixteen analyzed SpeB^A-^ variants of *emm*1 and *emm*12 isolates all acquired *covS* mutations during infection in mice. Thus, we conclude that both invasive and pharyngeal contemporary *emm*1 GAS isolates and *emm*12 GAS have a similar capacity to acquire CovRS mutations *in vivo*.

## Materials and Methods

### Declaration of ethical approval

All animal experimental procedures were carried out in strict accordance with the recommendations in the Guide for the Care and Use of Laboratory Animals of the National Institutes of Health [40]. The protocols for mouse experiments were approved by the Institutional Animal Care and Use Committee at MSU (Permit numbers: 2011–57 and 2014–45).

### GAS laboratory strains, clinical isolates, and M protein gene (*emm*) typing

M1T1 strain MGAS2221 [14] and its *cov*S deletion mutant, MGAS2221 Δ*covS* [16], have been previously described. A *rocA* deletion mutant of MGAS2221 (MGAS2221 Δ*rocA*) will be described elsewhere. One hundred seventy-six invasive GAS isolates from patients with NF and/or STSS were collected from 2010–2013, and their *emm* genotypes were determined by the *Streptococcus* Laboratory at the Centers for Disease Control and Prevention (CDC) (S1 Table). These CDC isolates were from 10 diverse geographic areas in the United States. Fifty GAS isolates (HMC isolates) were isolated from throat swabs obtained from patients with pharyngitis during the period of May-August 2014 by the Harborview Medical Center Clinical Microbiology Laboratory in Seattle, Washington (S2 Table). For these invasive isolates from CDC, we simply requested in 2013 the most recent isolates from patients with severe invasive infections of necrotizing fasciitis with or without streptococcal toxic shock syndrome (STSS) and STSS without necrotizing fasciitis. For the pharyngeal isolates, the Harborview Medical Center Clinical Microbiology Laboratory collected 50 pharyngitis isolates in 2014 without any selection criteria. The *emm* genotypes of the HMC pharyngeal isolates were determined by *emm* typing according to the CDC *emm* typing protocol [41] and by using the CDC *emm* database at http://www2a.cdc.gov/ncidod/biotech/strepblast.asp.

### Bacterial growth

GAS bacteria were statically grown at 37°C in 5% CO_2_ in Todd–Hewitt broth supplemented with 0.2% yeast extract (THY). Tryptose agar with 5% sheep blood and THY agar were used as solid media.

### PCR analysis of *speA* and *sda1* in clinical M1 isolates

The presence of the DNase *sda1* and exotoxin A (*speA*) genes in *emm1* clinical isolates was determined by the presence of PCR products using primer pairs 5’-ATTATTCACTCTGCTCTGACC-3’/5’-CCCTCTTTACCATTTAT-3’ and 5’-CTTGGTTGTTAGGTAGACTTC-3’/5’-ATCTCGCAAGAGGTATTTGC-3’, respectively.

### Selection of SpeB^A-^ variants in mice

Analysis of *in vivo* selection of SpeB^A-^ variants of clinical GAS isolates was performed as described previously [17]. More details regarding the experimental procedures and animal care are given below. Female, five-week old C57BL/6 mice used in the selection assay were bred at the Animal Resource Center at Montana State University (ARC) using breeding pairs of mice from the Jackson Laboratory (Bar Harbor, Maine). Adequate care of the animals used in experiments was provided by ARC in accordance with the standards incorporated in the Guide to the Care and Use of Laboratory Animals, 1996 edition (National Academy Press, Washington, D.C.). ARC, an 18,000-sq. ft facility, is fully accredited by the American Association for Accreditation of Laboratory Animal Care and is under the direction of a full-time, ACLAM-board-certified veterinarian. MSU complies with the NIH policy on animal welfare (letter of assurance filed), the Animal Welfare Act and all other applicable federal, state, and local laws. The facility is supplied with HEPA-filtered air and uses individually ventilated cages for mice.

GAS bacteria used in the selection assay were harvested from THY culture at the exponential growth phase and washed three times with pyrogen-free Dulbecco’s phosphate-buffered saline (DPBS) and resuspended in DPBS. Groups of 5 or 10 mice were subcutaneously inoculated for each strain with 0.2 ml of GAS suspension in DPBS with an OD_600_ of 0.9 after mice were anesthetized by isoflurane inhalation using an isoflurane vaporizer from VetEquip Inhalation Anesthesia Systems. Mice infected with GAS were monitored three times a day at about 8:00 am, 12:30 pm, and 16:30 pm. At day 4 after GAS inoculation, mice were euthanized by CO_2_ inhalation, which was done with a gradual fill method at a displacement rate of 30% CO_2_ of the chamber volume per minute, as recommended in The 2013 American Veterinary Medical Association Guidelines. SpeB^A+^ GAS isolates at the used dose caused ruffled fur but did not cause death at the endpoint of the experiment in this subcutaneous infection model.

Skin infection sites of the euthanized mice at day 4 after GAS inoculation were collected and homogenized in 1.0 ml DPBS using a Kontes pestle. The samples were then plated at 10^3^-, 10^4^-, and 10^5^-fold dilutions to obtain well isolated colonies. Forty-eight colonies for each mouse of each test strain were randomly picked, inoculated in 200 μl THY in 96-well plates, and cultured overnight at 37°C in 5% CO_2_. Three μl of 10% β-mercaptoethanol were added into each well, and the cultures were centrifuged in the plate at 3,500 rpm for 10 min. The supernatant samples, 15 μl each, were loaded into wells on casein gel plates and incubated at 37°C for 3 h. The casein gel plate with 96 wells was prepared as follows: 125 ml of heat-solubilized agarose and casein aqueous solution containing 0.38 g Tris base, 1.1 g NaCl, 0.84 g casein, and 1.25 g agarose was poured into the cover plate of a cell culture plate (Greiner Bio-One International Cat.-No. 665 180), and the tube bottom of a 96-well PCR plate was inserted into the casein solution during gel formation. The formation of a cloudy ring around wells due to casein hydrolysis was the indication of the presence of the SpeB activity in culture supernatant (SpeB^A+^), and the lack of the cloudy ring indicated lack of the SpeB activity in the samples (SpeB^A-^). The number of SpeB^A-^ colonies among 48 colonies from a mouse was counted to calculate the percentage of SpeB^A-^ variants among GAS recovered from each mouse.

### DNA sequencing

A DNA fragment containing the *covRS* genes was amplified from test strains using primers 5’-TCGCTAGAAGACTATTTGAC-3’ and 5’-TTCATGTCATCCATCATTGC-3’ and the Phusion high-fidelity PCR kit from New England BioLabs. DNA sequencing of the amplified PCR products was performed using the BigDye Terminator v3.1 Cycle Sequencing Kit and an Applied Biosystems 3130 genetic analyzer. Primers used for sequencing *covRS* were 5’-TCGCTAGAAGACTATTTGAC-3’, 5’-TTCATGTCATCCATCATTGC-3’, 5’-AACGGCTTCATCATATTTCC-3’, 5’-AAATCCACAAAACCGTTCAG-3’, 5’-TGATACACACGACCGATAG-3’, 5’-TTGATGACAGAAAGGGCAG-3’, 5’-TACGCGAACCATGTCTAAC-3’, and 5’-GTTGGGGTAAAGATGACAG-3’. DNA fragment containing the *rgg* gene was amplified and sequenced using primers 5’-GTAACAATAACCACATAGTAGGCG-3’ and 5’-TCGTCATTGCTTTTTATGATTTGTC-3’. DNA fragment in *emm*1 isolates containing the G/A polymorphism at base 989 of the *nga* gene was amplified using primers 5’-gaacagatgtgaaggttctgtg-3’ and 5’-gtctcttctagtgatacgatac-3’ and sequenced using primer 5’-GATTTGATGTTAGCTTTTGATGATG-3’. Sequence data were analyzed using the software Sequencer 5.1 from the Gene Codes Corporation.

### Analyses for SsE production

Relative levels of SsE in culture supernatants of parent isolates and their SpeB^A-^ variants were determined by measuring its platelet-activating factor acetylhydrolase activity using the 2-thio-PAF hydrolysis assay [42]. Briefly, 100 μl of the culture supernatant from the exponential growth phase (OD_600_ = ~0.4) was mixed with 100 μl of reactant solution containing 0.9 mM 2-thio-PAF and 1.3 mM 5,5'-dithiobis-(2-nitrobenzoic acid) at room temperature in wells of a 96-well plate. Absorbance change at 414 nm (ΔA_414_) as a measure of SsE-catalyzed 2-thio-PAF hydrolysis was recorded with time using a SPECTRA^Max^ 384 Plus spectrophotometer (Molecular Devices).

### Statistical analysis

**Fig 1:**
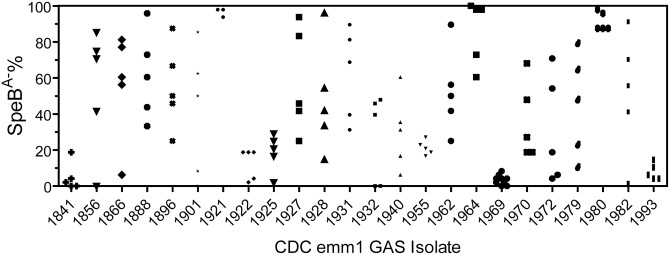
Selection of SpeB^A-^ variants of SpeB^A+^ invasive *emm*1 isolates in subcutaneous infection of mice. Five or 10 mice were subcutaneously inoculated with ~10^8^ cfu of each of the indicated 24 *emm*1 invasive isolates. Four days later, GAS bacteria were recovered from skin infection sites, and 48 colonies from each mouse were tested for SpeB activity in overnight culture supernatant by the casein hydrolysis assay. Presented are percentages of variants without detectable SpeB activity among the 48 colonies.

**Fig 2:**
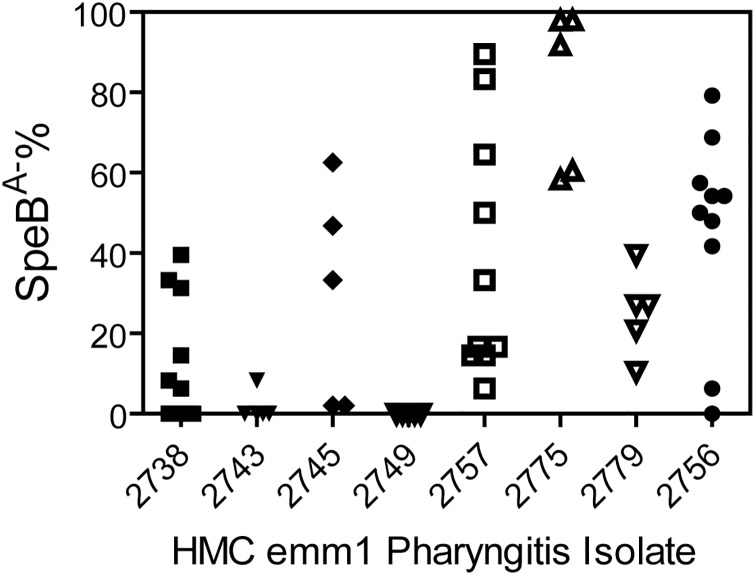
Selection of SpeB^A-^ variants of SpeB^A+^ *emm*1 pharyngeal isolates in mice. Shown are SpeB^A−^% among GAS bacteria recovered from skin infection sites of mice at day 4 after subcutaneous inoculation with ~10^8^ cfu of each of the indicated 8 *emm*1 pharyngeal isolates. The SpeB^A-^% values were determined as described in Fig 1 legend.

The GraphPad Prism 7 software program (Graph-Pad Software, Inc.) was used for statistical analyses. The data of *in vivo* selection for SpeB^A-^ variants in Figs 1 and 2 were analyzed using one way ANOVA Tukey’s multiple comparison test.

## Results

### Clinical invasive and pharyngeal isolates

The objective of this study was to determine whether pharyngeal *emm*1 GAS isolates are able to acquire CovRS mutations as readily as invasive M1 isolates *in vivo* and whether *emm*3, *emm*12, and *emm*28 GAS can acquire CovRS mutants in mouse infection. To achieve this objective, we obtained 176 invasive GAS isolates from the CDC in the United States from 2010 through early 2013 (S1 Table). The 176 GAS samples included 102 isolates from patients with NF only, 60 ones from patients with STSS only, and 14 isolates with both NF and STSS. The Harborview Medical Center Clinical Microbiology Laboratory (Seattle, Washington) (HMC) collected 50 GAS isolates from patients with pharyngitis in May-August 2014 for this study (S2 Table).

The 5 most prevalent *emm* genotypes of these invasive isolates were *emm*1 (31 isolates), *emm*89 (21 isolates), *emm*3 (16 isolates), *emm*12 (15 isolates), and *emm*28 (11 isolates) (Table 1). These were the most common *emm* types in the US at that time [37]. The 4 most prevalent *emm* genotypes among the pharyngeal isolates were *emm*1 (9 isolates or 18%), *emm*12 (8 isolates or 16%), *emm*89 (5 isolates or 10%), *emm*28 (4 isolates or 8%) (Table 2). Each of the *emm*2, *emm*3, and *emm*87 genotypes was represented by 3 pharyngeal isolates (6%). A limitation of the HMC GAS collection is its small size and origin from a single location. Nevertheless, it is reasonable to conclude that the *emm*1 genotype appears to be prevalent among both invasive and pharyngeal GAS isolates and that the prevalent *emm* genotypes in severe invasive infections are similar to the prevalent *emm* genotypes of circulating pharyngeal GAS strains, which is discussed in more details in Discussion.

**Table 1:** Distribution of *emm* genotypes and %SpeB^A-^ among the CDC Invasive GAS Isolates.

*emm* genotype	No. of Isolates	No. of SpeB^A-^ isolates	% SpeB^A-^ isolates
1	31	7	25.8
89	21	17	81.0
3	16	10	62.5
12	15	3	20.0
28	11	6	54.5
4	8	2	25.0
118	8	4	50.0
11	6	1	16.7
59	6	1	16.7
75	5	2	40.0
2	4	1	25.0
6	4	0	0.0
82	4	1	25.0
87	4	1	25.0
44	3	0	0.0
77	3	0	0.0
22	3	1	33.3
81	3	0	0.0
92	3	0	0.0
5	2	0	0.0
9	2	1	50.0
58	2	0	0.0
73	2	0	0.0
76	2	1	50.0
78	2	1	50.0
18	1	1	100
41	1	1	100
49	1	1	100
53	1	1	100
113	1	1	100
114	1	0	0.0
total	176	66	37.5

**Table 2:** Distribution of *emm* genotypes and %SpeB^A-^ among the HMC pharyngeal isolates.

*emm* genotype	No. of isolates	No. of SpeB^A-^ isolates	% SpeB^A-^ isolates
1	9	1	11.1
12	8	1	12.5
89	5	0	0.0
28	4	0	0.0
2	3	0	0.0
3	3	1	33.3
87	3	0	0.0
9	2	1	50.0
4	1	0	0.0
6	1	1	100
22	1	1	100
48	1	0	0.0
58	1	0	0.0
59	1	0	0.0
75	1	0	0.0
77	1	0	0.0
81	1	0	0.0
82	1	0	0.0
85	1	0	0.0
111	1	0	0.0
238	1	0	0.0
total	50	6	12.0

### Presence of *sda*1, *speA*, *and nga* 989G allele in all *emm*1 isolates

To determine whether all the *emm*1 isolates in both collections belonged to the M1T1 subclone, *emm*1 isolates were analyzed by PCR using *sda1*- and *speA*-specific primers, and all strains generated PCR products of expected sizes. Thus, all the *emm*1 isolates carried the *sda1* and *speA* genes. It has been reported that isolates of the M1T1 clone have a replacement of a 36-kb, *slo*- and *nga*-containing chromosomal region of pre-1980 *emm*1 GAS with that of serotype *emm*12 GAS, which results in enhanced expression of SLO and NADase and has the 989G allele in the NADase gene *nga* [6–9]. We attempted to compare SLO levels in mid-exponential growth phase culture supernatants of SpeB^A+^ *emm*1 isolates, M1T1 strain MGAS2221 (positive control), and SF370 (a pre-1980 M1 strain and negative control) by Western blotting using SLO-specific antibodies. However, we could not consistently distinguish SF370 from MGAS2221 or SpeB^A+^ *emm*1 isolates on the basis of SLO levels. Thus, we determined the polymorphism at base 989 of the *nga* gene, and all the *emm*1 isolates had the 989G allele. Based on these results, all of the *emm*1 isolates in the two collections are most likely similar to the contemporary Finland pharyngeal *emm*1 isolates that are closely related to MGAS5005 [9], a representative strain of the currently pandemic M1T1 clone.

### SpeB production in clinical invasive and pharyngeal isolates

Next, SpeB protease activity in the supernatants of overnight cultures of the clinical isolates was assayed by casein hydrolysis. Overall, 37.5% of the 176 invasive isolates were SpeB^A-^. SpeB^A+^ isolate samples did not contain SpeB^A-^ bacteria as determined by checking SpeB activity of 5 or more independent colonies of each sample. Among the 5 most frequent *emm* genotypes of the invasive isolates, about 20% of *emm*1 and *emm*12 isolates, 60% of *emm*3 and *emm*28 isolates, and 80% of 21 invasive *emm*89 isolates were SpeB^A-^ (Table 1). The SpeB^A-^ percentage of the invasive *emm*1 isolates is slightly higher than reported for a larger invasive *emm*1 GAS collection [38]; however, the percentage of our invasive *emm*89 isolates that were SpeB^A-^ is 4 times higher than observed in invasive *emm*89 isolates in the earlier study [38]. As our 5 *emm*89 pharyngeal isolates were all SpeB^A+^, the association of the SpeB^A-^ phenotype with *emm*89 NF and/or STSS isolates appears to be genuine. The percentages of SpeB^A-^ isolates among NF, STSS, and NF/STSS isolates were 39.2%, 33.3%, and 42.8%, respectively. Thus, isolates from these three different forms of severe invasive infection exhibit similar percentages of isolates with the SpeB^A-^ phenotype.

Six of the 50 HMC pharyngeal isolates were SpeB^A-^, including 1 isolate each from the *emm*1, *emm*3, *emm*6, *emm*9, *emm*12, and *emm*22 genotypes (Table 2). Notably, all 5 *emm*89 isolates and 8 out of 9 *emm*1 isolates were SpeB^A+^. Thus, pharyngeal *emm*1 and *emm*89 isolates appear to have fewer percentages of isolates with the SpeB^A-^ phenotype than NF and/or STSS isolates.

### Selection of SpeB^A-^ variants of invasive and pharyngeal *emm*1 SpeB^A+^ isolates

The screening of invasive and pharyngeal isolates by the SpeB activity assay identified 24 invasive and 8 pharyngeal *emm*1 SpeB^A+^ isolates (Table 3). These isolates allowed us to determine whether invasive and pharyngeal *emm*1 GAS isolates have a different tendency to acquire CovRS mutations *in vivo*. The study was mainly the comparison of pharyngeal *emm*1 isolates with severe invasive *emm*1 isolates in the capacity to acquire CovRS mutants *in vivo*, and 19 of the 31 severe invasive *emm*1 isolates were from patients with necrotizing fasciitis. Thus, both pharyngeal and subcutaneous infection models should be relevant for the purpose. We had preliminary data for *in vivo* selection of MGAS2221 *covRS* mutants in intranasal infection model. However, the intranasal model is more technically challenging because of the presence of non-GAS bacteria. Thus, we chose the model of subcutaneous infection of mice. We used the SpeB activity assay to identify SpeB^A-^ variants among isolates that were recovered from skin infection sites in mice on day 4 after inoculation of each SpeB^A+^ *emm*1 GAS isolate. SpeB^A-^ variants were detected at skin infection sites for all 24 invasive SpeB^A+^ *emm*1 isolates (Fig 1). Twenty-one of them had mean SpeB^A-^% values ranging from 12.5% to 96.5%, and 3 others had low mean SpeB^A-^% values ranging from 3.5% to 7.9% (Table 3). SpeB^A-^ variants were detected at skin infection sites for 7 of the 8 SpeB^A+^ pharyngeal *emm*1 isolates (Fig 2). Six had mean SpeB^A-^% values from 13.3% to 81.2%, and one had 1.7% SpeB^A-^ variants (Table 3). One pharyngitis isolate had no detectable SpeB^A-^ variants. Thus, like contemporary invasive *emm*1 isolates, the majority of contemporary pharyngeal *emm*1 GAS isolates are able to give rise to SpeB^A-^ variants *in vivo*.

**Table 3:** Average percentages of SpeB^A-^ variants selected from invasive and pharyngeal *emm*1 SpeB^A+^ isolates and P values in statistical analysis.

Strain number	Infection	Avg. SpeB^A-^ variants (%)^a^	Group	Significant P value^b^
1921	Invasive	96.5		iG1 vs iG2: <0.0001; iG1 vs iG4: ≤0.0372; iG1 vs pG2: <0.0001; iG1 vs pG4: ≤0.0216; iG2 vs pG1: ≤0.0003; iG3 vs iG2: <0.05
1964	Invasive	85.8	iG1	
1980	Invasive	91.3		
1841	Invasive	5.0		
1969	Invasive	3.5	iG2	
1993	Invasive	7.9		
1856	Invasive	54.6		
1866	Invasive	56.2		
1888	Invasive	61.3		
1896	Invasive	55.0		
1901	Invasive	41.2		
1927	Invasive	57.9	iG3	
1928	Invasive	47.9		
1931	Invasive	62.1		
1962	Invasive	52.5		
1970	Invasive	36.1		
1979	Invasive	45.0		
1982	Invasive	52.5		
1922	Invasive	12.5		
1932	Invasive	26.7		
1925	Invasive	18.8	iG4	
1940	Invasive	30.0		
1955	Invasive	21.3		
1972	Invasive	30.8		
2775	Pharyngeal	81.2	pG1	pG1 vs pG2: <0.0001; pG1 vs pG3: ≤0.0119; pG1 vs pG4: ≤0.0059; pG2 vs pG3: ≤0.0405
2743	Pharyngeal	1.7	pG2	
2749	Pharyngeal	0		
2756	Pharyngeal	46.0	pG3	
2757	Pharyngeal	39.0		
2745	Pharyngeal	29.4		
2738	Pharyngeal	13.3	pG4	
2779	Pharyngeal	25.0		

^*a*^The average SpeB^A-^% values were calculated from the data in Figs 1 and 2.

^*b*^P values were from the analysis of the SpeBA-% data using one way ANOVA Tukey’s multiple comparison test

### Significant difference among *emm*1 isolates in the capacity to acquire SpeB^A-^ variants in murine subcutaneous infection

Isolates of both the invasive and pharyngeal *emm*1 collections had variable average SpeB^A-^% value in the *in vivo* selection for SpeB^A-^ variants in mice. Statistical analysis of these data using one way ANOVA Tukey’s multiple comparison test could divide the isolates into four groups on the basis of significant difference in the SpeB^A-^% data for both invasive (iG1-iG4) and pharyngeal (pG1-pG4) isolates (Table 3). The iG1 and pG1 isolates had >80% of average SpeB^A-^% and were significantly different in SpeB^A-^% from iG2 and pG2 with average SpeB^A-^% of <10% (P <0.0001), and the average SpeB^A-^% values of the iG4 and pG4 isolates ranging from 10% to 30% were significantly different from those of the iG1 and pG1 isolates (P <0.05). Both iG3 and pG3 had average values of SpeB^A-^% ranging from 30% to 60% and were not significantly different from isolates in the iG, iG2, pG1, and pG2 groups. Thus, some isolates in both invasive and pharyngeal *emm*1 isolates have significantly higher capacity to acquire SpeB^A-^ variants than others in the same categories.

### CovS mutations and enhanced SsE expression in *emm*1 SpeB^A-^ variants selected in mouse infection

**Fig 3:**
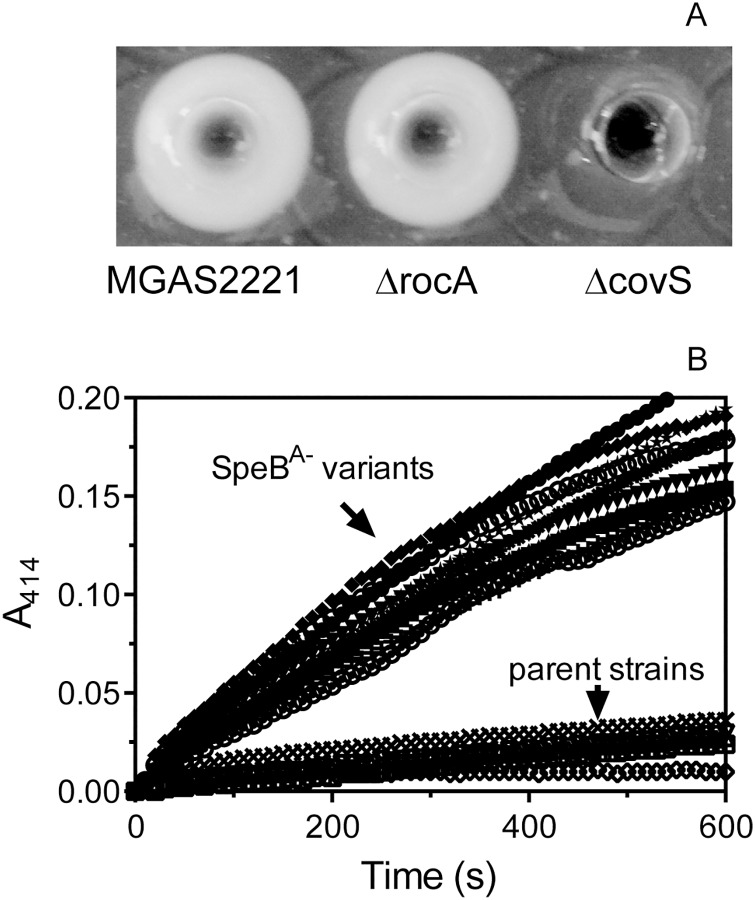
Evidence for CovRS mutations as the basis for the SpeB^A-^ phenotype of variants selected in subcutaneous infection of mice. (A) MGAS2221 and its *rocA* deletion mutant, but not its *covS* deletion mutant, had SpeB protease activity in overnight culture supernatant as determined by the casein hydrolysis assay. (B) Levels of SsE platelet-activating factor acetylhydrolase activity in culture supernatant at the exponential growth phase of 10 SpeB^A-^ variants and their 5 *emm*1 SpeB^A+^ parent strains listed in Table 4. The SsE activity was measured using the colorimetric assay as described in the Methods.

Although we have reported that the SpeB^A-^ phenotype is a reliable indicator for CovRS mutations of MGAS2221 in subcutaneous infection of mice [17], CovRS mutations may not be the only cause of the SpeB^A-^ phenotype. RocA has been shown to function through CovR, and *rocA* deletion enhances expression of CovRS-regulated virulence genes [43]. Thus, we determined whether *rocA* mutations are a cause of the SpeB^A-^ phenotype by comparing the SpeB activity in culture supernatant of M1T1 strain MGAS2221 and its Δ*rocA* and Δ*covS* mutants. MGAS2221 Δ*covS* was SpeB^A-^ but both MGAS2221 Δ*rocA* and MGAS2221 were SpeB^A+^ (Fig 3A). Thus, *rocA* mutations are not a cause of the SpeB^A-^ phenotype. Another possible common cause of the SpeB^A-^ variants is mutations in the *rgg* gene [38,39,44]. We sequenced the *covRS* and *rgg* genes in 5 *emm*1 invasive SpeB^A+^ isolates and 2 SpeB^A-^ variants of each of them selected in skin infection. The 5 clinical isolates all had same *covRS* and *rgg* sequences with MGAS2221, and all 10 SpeB^A-^ variants had missense mutations or indels of *covS* but have wild-type *rgg* (Table 4). Being consistent with the previous findings that natural CovRS mutations enhance SsE expression [16,29,33], all the SpeB^A-^ variants had higher levels of SsE PAF acetylhydrolase activity than their parent strains in culture supernatant at the exponential growth phase (Fig 3B). Thus, SpeB^A-^ variants of *emm1* GAS selected in subcutaneous infection in mice are caused primarily by CovRS mutations and have enhanced virulence gene expression.

**Table 4:** *covS* mutations of SpeB^A-^ variants of *emm1* and *emm12* GAS in subcutaneous mouse infection.

Strain^a^	Parent strain	*emm type*	*covS* mutation^b^	Mutated CovS	*rgg* mutation^b^
2253	1841	1	C685T	Arg229Cys	No
2254	1841	1	A insertion at 1048	Truncated	No
2134	1856	1	Δ1341A	Truncated	No
2135	1856	1	C59T	Ser20Phe	No
2144	1866	1	Δ^83^T	Truncated	No
2145	1866	1	Δ^83^T	truncated	No
2154	1888	1	Δ^1215^GAAAA	truncated	No
2155	1888	1	Δ^1215^GAAAA	truncated	No
2258	1896	1	C737T	Ser246Leu	No
2259	1896	1	Δ^83^T	Truncated	No
3158	1845	12	G1279T	Gly427Cys	No
3159	1845	12	Δ^83^T	truncated	No
3160	1845	12	A980T	Asp327Val	No
3161	1845	12	T1391C	Ile464Thr	No
3162	1867	12	Δ^83^T	truncated	No
3164	1867	12	C104T	Thr35Ile	No

^*a*^Two SpeB^A-^ variants from the same mouse derived from each indicated parent strain except that 4 variants derived from strain 1845 were from two mice.

^*b*^Mutations refer to genetic changes in comparison with the wt *covS* and *rgg* sequences in the parent strains.

### Selection of CovRS mutants of *emm*12 GAS in subcutaneous mouse infection

**Fig 4:**
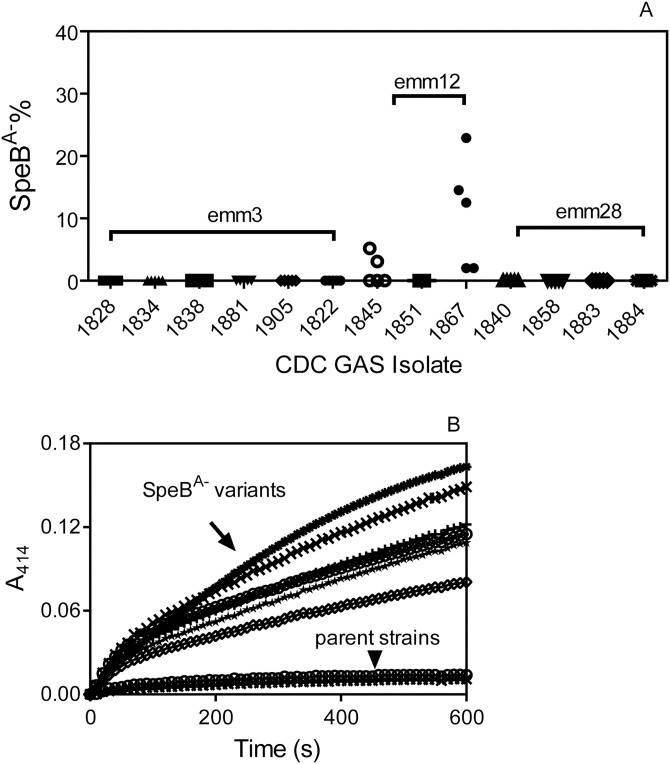
Selection of CovRS mutants of *emm*12 GAS in subcutaneous mouse infection. (A) The capacity of SpeB^A+^ *emm*3, *emm*12, and *emm*28 invasive isolates to give rise to SpeB^A-^ variants in mice. Shown are SpeB^A−^% among GAS bacteria recovered from skin infection sites of mice at day 4 after subcutaneous inoculation with ~10^8^ cfu of each of the indicated 6 *emm*3, 3 *emm*12, and 4 *emm*28 invasive isolates. (B) Levels of SsE platelet-activating factor acetylhydrolase activity in culture supernatant at the exponential growth phase of 6 SpeB^A-^ variants and their 2 *emm*12 SpeB^A+^ parent strains listed in Table 4.

To determine whether *emm*3, *emm*12, and *emm*28 GAS has the capacity to acquire SpeB^A-^ mutations in mouse infection, we tested 6 *emm*3, 3 *emm*12, and 4 *emm*28 SpeB^A+^ invasive isolates for selection of SpeB^A-^ variants during cutaneous infection in mice. No SpeB^A-^ variants were detected at day 4 after infection with the *emm*3 and *emm*28 isolates (Fig 4A). However, 10.8% of recovered M12 isolate 1867 were SpeB^A-^, and isolates recovered from *emm*12 strain 1845 infection contained 1.7% SpeB^A-^ variants (Fig 4A). Four SpeB^A-^ variants of GAS1845 and 2 SpeB^A-^ variants of GAS1867 all had *covS* mutation or deletion and lacked *rgg* mutation compared with the parent strains (Table 4). Like the *emm*1 SpeB^A-^ variants, all these *emm*12 SpeB^A-^ variants had enhanced SsE PAF acetylhydrolase activity in their culture supernatant at the exponential growth phase (Fig 4B). Thus, arising of *emm*12 GAS CovRS mutants is demonstrated in subcutaneous infection of mouse.

## Discussion

The main objective of this study was to test the hypothesis that pharyngeal *emm*1 GAS isolates have less propensity to acquire CovRS mutations *in vivo* than invasive *emm*1 isolates, as a possible explanation for the rarity of severe invasive GAS infections caused by pandemic M1T1 GAS. Our experimental observations do not support this hypothesis, as contemporary *emm*1 isolates from pharyngitis patients and patients with NF and/or STSS exhibit similar capacity to acquire CovRS mutations following subcutaneous infection of mice. Thus, the explanation for the rarity of severe invasive GAS infections remains to be fully elucidated. We also showed that contemporary *emm*1 isolates from both pharyngitis and severe invasive infections can have significantly different propensity to acquire CovRS mutations. In addition, we found that *emm*12, but not *emm*3 and *emm*28, GAS isolates can acquire CovRS mutations in subcutaneous infection of mice. The findings provides information for the understanding of the arising of GAS mutants in a hostile environment.

Although contemporary *emm*1 isolates belong to the M1T1 clone, they have single nucleotide polymorphisms [9,14]. It is possible that some M1T1 GAS bacteria acquire rare genetic alterations that render them to selection for CovRS mutations, causing rare severe invasive infections. However, this possibility has been excluded by our finding that pharyngeal *emm*1 isolates have the capacity to acquire CovRS mutations during mouse infection at frequencies similar to those of invasive *emm*1 isolates. Nevertheless, our data are consistent with the consensus notion in the field that contemporary *emm*1 GAS strains cause severe invasive infections in only a small fraction of the population, suggesting that additional bacterial genetic determinants and/or host factors are involved.

As in a recent analysis of SpeB production by invasive GAS isolates, the majority (74.2%) of the CDC *emm*1 isolates were found to be SpeB^A+^ [38]. The prevalence of SpeB^A-^ isolates is similar to that of SpeB^A-^ variants identified on day 4 after murine infection with SpeB^A+^ *emm*1 isolates. Indeed, mixed GAS populations with or without CovRS mutations are observed in patients [45]. The likelihood to isolate a CovRS mutant depends on where and when isolates are recovered from the host [5]. Thus, the frequency of SpeB^A-^ and SpeB^A+^ phenotypes among clinical invasive GAS isolates should be interpreted with caution. Mixed GAS populations with or without CovRS mutations are also consistent with the suggestion that CovRS mutations arise during human infection with GAS carrying wild-type CovRS and are not transmissible [22]. The inability of CovRS mutants to transmit suggests a "source-sink" dynamic in which GAS with wild-type CovRS has an advantage in transmission and pharyngeal infection while GAS with CovRS mutations contributes to rarer episodes of severe invasive disease in susceptible population.

The pharyngeal isolates in this study were from a single location whereas the CDC invasive isolates represent diverse geographic areas in the United States. Whether our comparison is relevant depends on whether the pharyngeal *emm*1 isolates in Seattle are similar to those in the other geographic areas in the United States. It is well established that contemporary *emm*1 GAS strains belonged to the invasive M1T1 subclone emerged in the 1980s and have essentially displaced antecedent *emm*1 strains across a broad geographic region [3,4,9]. Contemporary severe and non-severe invasive *emm*1 isolates in the United States are genetically related to the invasive M1T1 clone [9]. Contemporary pharyngeal *emm*1 isolates in the United States must be dominantly related to the invasive M1T1 clone because there are evidences for the origination of invasive GAS strains from pharyngitis strains [34,46]. This possibility is supported by the fact that contemporary pharyngeal and invasive *emm*1 strains in Canada and Finland were related to the invasive M1T1 clone [9]. All the pharyngeal and invasive *emm*1 isolates in this study carry the *sda*1 and *speA* genes and the *nga* 989G allele, consistent with the invasive M1T1 clone. These data and the previous findings support that *emm*1 pharyngeal GAS strains in the United States are dominantly related to the M1T1 subclone. Thus, the geographic limitation in our analyses unlikely invalidates our conclusion that contemporary pharyngeal and invasive *emm*1 isolates have a similar capacity to acquire CovRS mutations *in vivo*.

Invasive GAS isolates frequently have CovRS mutations resulting in a SpeB^A-^phenotype [20,21,38]. However, the selection of SpeB^A-^ variants has been previously demonstrated in mouse infections only for M1T1 GAS [8,13,14,17,31–33]. Neutrophils are required for *in vivo* selection of M1T1 GAS SpeB^A-^ variants [17]. The capsule and M protein are critical for the selection of SpeB^A-^ variants of M1T1 GAS [32]. However, capsule and M protein are critical virulence factors for both contemporary and pre-1980 *emm*1 GAS strains. Reports are conflicting as to whether Sda1 is critical for the selection of SpeB^A-^ variants of invasive M1T1 GAS [31,33]. Thus, the critical trigger for *in vivo* selection of M1T1 SpeB^A-^ variants is presently unknown. The present study shows that selection of CovRS mutants of *emm*12 GAS isolates can also be demonstrated in mice. We also showed that some *emm*1 isolates are overpopulated by emerged CovRS mutants within a few days while others accumulate small percentages of CovRS mutants at the same time point in subcutaneous infection of mice. Further characterization of the selection of CovRS mutants of *emm*12 GAS and elucidation of the basis for the differential propensity of contemporary *emm*1 GAS to acquire CovRS mutants in mouse infection may provide additional clues regarding the trigger for *in vivo* selection of GAS CovRS mutations.

No SpeB^A-^ variants were detected after cutaneous infection with SpeB^A+^ *emm*3 isolates. One possible reason is that *emm3* isolates have a natural *rocA* mutation that enhances expression of CovRS-controlled virulence factors [43] and thus alleviates the selective pressure for CovRS mutants. However, this possibility is not consistent with the observation that invasive *emm*3 isolates are more likely to contain CovRS mutations than pharyngeal *emm*3 isolates [24]. Furthermore, even in a *rocA* null background, CovRS mutations enhance the expression of virulence genes and critically contribute to *emm*3 GAS virulence [21]. An alternative possibility is that the screening of M3 GAS by the SpeB activity assay failed to pick up certain CovRS mutations. For example, the CovS^G457V^ point mutation of invasive M3 isolate MGAS315 enhances expression of virulence genes and critically contribute to its virulence; however, this point mutation does not cause a SpeB^A-^ phenotype in MGAS315 [19]. It is also possible that human infections have a mechanism for the selection of *emm*3 GAS CovRS mutants that is not mimicked in the murine skin infection model.

It is interesting that 17 of 21 invasive *emm*89 isolates (80%) are SpeB^A-^ while all 5 pharyngeal *emm*89 isolates are SpeB^A+^. It has been reported that 20.7% of invasive *emm*89 isolates are SpeB^A-^ [38], and these *emm*89 isolates belong to three major phylogenetic groups [11]. GAS in one of the tree groups lacks the *hasABC* capsule biosynthesis locus and has emerged as a frequent cause of severe invasive infections since 2000 [10–12]. The unusually high prevalence of the SpeB^A-^ phenotype in CDC *emm*89 invasive isolates could be due to an enhanced selection pressure for acapsular *emm*89 GAS strains, leading to a propensity to cause necrotizing fasciitis and toxic shock syndrome.

Kazmi *et al*. first reported SpeB^A+^-to-SpeB^A-^ phase shift of M1T1 GAS in mouse infection [47]. A *covS* mutant of M1T1 GAS arisen in mice confers the SpeB^A-^ phenotype [14]. The regulator *rgg* also regulates *speB* [48]. The SpeB^A-^ phenotype has been detected in many clinical isolates, and clinical SpeB^A-^ isolates are associated dominantly with mutations in *rgg* and *covS* and less frequently with *covR* mutations [38]. Surprisingly, we only detected mutations of *covS* but not *rrg* in SpeB^A-^ variants of *emm*1 and *emm*12 isolates in subcutaneous infection of mice in this study. Previous studies also reported that SpeB^A-^ variants of *emm1* GAS selected in mouse infection are associated with *covRS* mutations [8,13,14,15,17,31–33]. All these data resonate the lack of the selection of SpeB^A-^ variants of *emm*3 GAS in subcutaneous infection of mice whereas *covRS* mutations are frequent in *emm*3 clinical isolates [21]. These findings suggest that different GAS strains may be subject to different stresses in different infections and that mutations of *covRS* and *rgg* confer different fitness advantage in serotype-dependent and infection-dependent ways.

## Conclusions

We conclude that contemporary invasive and pharyngeal *emm*1 GAS isolates have a similar capacity to acquire CovRS mutations *in vivo*. We also conclude that *emm*2, but not *emm*3 and *emm*28, GAS can also readily acquire CovRS mutants in subcutaneous infection of mice.

## Supporting Information

S1 Table Serotype, Infection, and SpeB Secretion of CDC Invasive Isolates. (DOCX)

S2 Table *emm* Genotype and SpeB Secretion of HMC Pharyngitis Isolates. (DOCX)
