# Supplementary material for: Contemporary Pharyngeal and Invasive emm1 and Invasive emm12 Group A Streptococcus Isolates Exhibit Similar In Vivo Selection for CovRS Mutants in Mice
Source: PLoS One. 2016 Sep 9;11(9):e0162742. doi: 10.1371/journal.pone.0162742 (PMC5017694; doi:10.1371/journal.pone.0162742)
Supplement: S1 Table — (DOCX) [file pone.0162742.s001.docx]

S1 Table. Serotype, Infection, and SpeB Secretion of CDC Invasive Isolates*^a^*

| Strain # | CASPIRID | Year | *emm* type | Infection | | SpeB  Activity |
| --- | --- | --- | --- | --- | --- | --- |
|  |  |  |  | NF | STSS |  |
| 1824 | ABC020051519 | 2010 | 89 | + | + | - |
| 1825 | ABC020051160 | 2010 | 4 | - | + | - |
| 1826 | ABC020052554 | 2010 | 11 | + | + | + |
| 1827 | ABC020052556 | 2010 | 28 | - | + | - |
| 1828 | ABC020052558 | 2010 | 3 | - | + | + |
| 1830 | ABC020051679 | 2010 | 5 | - | + | + |
| 1831 | ABC020051702 | 2010 | 11 | - | + | + |
| 1832 | ABC020051709 | 2010 | 5 | - | + | + |
| 1833 | ABC020051959 | 2010 | 3 | - | + | - |
| 1834 | ABC020052023 | 2010 | 3 | - | + | + |
| 1835 | ABC020052034 | 2010 | 22 | - | + | + |
| 1836 | ABC020052131 | 2010 | 87 | - | + | + |
| 1837 | ABC020055937 | 2011 | 82 | + | - | + |
| 1838 | ABC020055975 | 2011 | 3 | - | + | + |
| 1839 | ABC020056022 | 2011 | 2 | + | - | + |
| 1840 | ABC020056103 | 2011 | 28 | - | + | + |
| 1841 | ABC020056115 | 2011 | 1 | + | + | - |
| 1842 | ABC020056140 | 2011 | 92 | + | - | + |
| 1843 | ABC020056170 | 2011 | 6 | + | - | + |
| 1844 | ABC020056742 | 2011 | 4 | + | - | + |
| 1845 | ABC020056755 | 2011 | 12 | + | - | + |
| 1846 | ABC020056767 | 2011 | 12 | + | - | - |
| 1847 | ABC020056774 | 2011 | 118 | + | - | + |
| 1848 | ABC020056776 | 2011 | 77 | + | - | + |
| 1849 | ABC020056797 | 2011 | 118 | + | - | - |
| 1850 | ABC020056895 | 2011 | 76 | + | - | + |
| 1851 | ABC020057016 | 2011 | 12 | - | + | + |
| 1852 | ABC020057019 | 2011 | 12 | - | + | + |
| 1853 | ABC020057051 | 2011 | 3 | - | + | - |
| 1854 | ABC020057109 | 2011 | 73 | + | - | + |
| 1855 | ABC020057128 | 2011 | 78 | + | - | - |
| 1856 | ABC020057170 | 2011 | 1 | - | + | + |
| 1857 | ABC020057200 | 2011 | 12 | + | - | + |
| 1858 | ABC020057304 | 2011 | 28 | + | - | + |
| 1859 | ABC020057305 | 2011 | 1 | + | + | - |
| 1860 | ABC020057318 | 2011 | 76 | + | - | - |
| 1861 | ABC020057325 | 2011 | 89 | + | - | - |
| 1862 | ABC020057331 | 2011 | 28 | + | - | - |
| 1863 | ABC020057351 | 2011 | 89 | + | - | + |
| 1864 | ABC020057504 | 2011 | 75 | + | - | + |
| 1865 | ABC020057547 | 2011 | 11 | - | + | - |
| 1866 | ABC020057548 | 2011 | 1 | - | + | + |
| 1867 | ABC020057550 | 2011 | 12 | + | - | + |
| 1868 | ABC020057688 | 2011 | 12 | + | - | - |
| 1869 | ABC020057692 | 2011 | 81 | + | - | + |
| 1870 | ABC020057693 | 2011 | 92 | + | - | + |
| 1971 | ABC020057744 | 2011 | 77 | + | - | + |
| 1872 | ABC020057769 | 2011 | 89 | + | + | - |
| 1873 | ABC020057932 | 2011 | 89 | + | - | - |
| 1874 | ABC020057994 | 2011 | 22 | + | - | - |
| 1875 | ABC020052189 | 2010 | 118 | - | + | - |
| 1876 | ABC020052287 | 2010 | 4 | + | + | + |
| 1877 | ABC020052305 | 2010 | 3 | - | + | - |
| 1878 | ABC020053440 | 2010 | 3 | - | + | - |
| 1879 | ABC020053958 | 2010 | 114 | - | + | + |
| 1880 | ABC020053992 | 2010 | 3 | - | + | - |
| 1881 | ABC020054353 | 2010 | 3 | - | + | + |
| 1882 | ABC020054522 | 2010 | 2 | - | + | + |
| 1883 | ABC020054652 | 2010 | 28 | + | + | + |
| 1884 | ABC020058039 | 2011 | 28 | + | - | + |
| 1885 | ABC020058050 | 2011 | 12 | + | - | - |
| 1886 | ABC020058056 | 2011 | 53 | - | + | - |
| 1887 | ABC020058125 | 2011 | 11 | + | - | + |
| 1888 | ABC020058255 | 2011 | 1 | + | + | + |
| 1889 | ABC020058339 | 2011 | 87 | + | - | + |
| 1890 | ABC020058355 | 2011 | 2 | + | - | - |
| 1891 | ABC020058380 | 2011 | 28 | + | + | - |
| 1892 | ABC020058443 | 2011 | 58 | + | - | + |
| 1893 | ABC020058539 | 2011 | 77 | + | - | + |
| 1894 | ABC020058559 | 2011 | 89 | + | - | - |
| 1895 | ABC020058644 | 2011 | 59 | + | - | + |
| 1896 | ABC020058758 | 2012 | 1 | - | + | + |
| 1897 | ABC020058759 | 2011 | 12 | + | - | + |
| 1898 | ABC020058875 | 2011 | 82 | + | - | + |
| 1899 | ABC020059290 | 2011 | 118 | + | - | + |
| 1900 | ABC020059511 | 2011 | 118 | + | - | + |
| 1901 | ABC020059526 | 2012 | 1 | - | + | + |
| 1902 | ABC020059554 | 2012 | 1 | - | + | - |
| 1903 | ABC020059568 | 2011 | 82 | + | - | - |
| 1904 | ABC020059470 | 2011 | 22 | + | - | + |
| 1905 | ABC020051269 | 2010 | 3 | - | + | + |
| 1906 | ABC020054973 | 2011 | 12 | - | + | + |
| 1907 | ABC020055005 | 2010 | 11 | - | + | + |
| 1908 | ABC020055018 | 2011 | 3 | + | - | - |
| 1909 | ABC020055218 | 2011 | 6 | + | - | + |
| 1910 | ABC020055221 | 2011 | 78 | + | - | + |
| 1911 | ABC020055395 | 2011 | 89 | - | + | - |
| 1912 | ABC020055561 | 2011 | 28 | - | + | + |
| 1913 | ABC020055563 | 2011 | 18 | - | + | - |
| 1914 | ABC020055614 | 2011 | 3 | + | - | - |
| 1915 | ABC020055661 | 2011 | 44 | + | - | + |
| 1916 | ABC020055664 | 2011 | 44 | + | - | + |
| 1917 | ABC020055718 | 2011 | 4 | + | - | + |
| 1918 | ABC020055734 | 2011 | 12 | + | - | + |
| 1919 | ABC020055744 | 2011 | 6 | + | - | + |
| 1920 | ABC020059655 | 2011 | 12 | + | - | + |
| 1921 | ABC020059984 | 2012 | 1 | + | - | + |
| 1922 | ABC020060002 | 2011 | 1 | + | + | + |
| 1923 | ABC020060200 | 2012 | 75 | - | + | - |
| 1924 | ABC020060216 | 2012 | 44 | - | + | + |
| 1925 | ABC020060221 | 2012 | 1 | - | + | + |
| 1926 | ABC020060251 | 2011 | 75 | - | + | + |
| 1927 | ABC020060255 | 2012 | 1 | + | - | + |
| 1928 | ABC020060258 | 2012 | 1 | - | + | + |
| 1929 | ABC020060264 | 2012 | 82 | - | + | + |
| 1930 | ABC020060282 | 2012 | 2 | + | - | + |
| 1931 | ABC020060302 | 2012 | 1 | + | - | + |
| 1932 | ABC020060303 | 2012 | 1 | + | - | + |
| 1933 | ABC020060310 | 2012 | 59 | + | - | - |
| 1934 | ABC020060451 | 2011 | 49 | + | - | - |
| 1935 | ABC020060464 | 2012 | 1 | - | + | - |
| 1936 | ABC020060528 | 2012 | 89 | + | - | - |
| 1937 | ABC020060604 | 2012 | 4 | - | + | - |
| 1938 | ABC020060692 | 2012 | 4 | + | - | + |
| 1939 | ABC020060710 | 2012 | 1 | + | - | - |
| 1940 | ABC020060712 | 2012 | 1 | - | + | + |
| 1941 | ABC020060734 | 2012 | 1 | + | - | - |
| 1942 | ABC020060786 | 2012 | 89 | + | - | - |
| 1943 | ABC020060793 | 2012 | 3 | - | + | - |
| 1944 | ABC020060795 | 2012 | 75 | + | + | - |
| 1945 | ABC020060796 | 2012 | 89 | - | + | - |
| 1946 | ABC020060816 | 2012 | 1 | + | - | - |
| 1947 | ABC020060826 | 2012 | 3 | + | - | - |
| 1948 | ABC020061050 | 2012 | 113 | - | + | - |
| 1949 | ABC020061108 | 2012 | 28 | + | - | - |
| 1950 | ABC020061119 | 2012 | 89 | - | + | - |
| 1951 | ABC020061138 | 2012 | 73 | - | + | + |
| 1952 | ABC020061140 | 2012 | 59 | + | - | + |
| 1953 | ABC020061295 | 2012 | 89 | - | + | + |
| 1954 | ABC020061407 | 2012 | 1 | + | - | - |
| 1955 | ABC020061465 | 2012 | 1 | - | + | + |
| 1956 | ABC020061554 | 2012 | 118 | + | - | + |
| 1957 | ABC020061569 | 2012 | 89 | + | - | - |
| 1958 | ABC020061593 | 2012 | 59 | + | - | + |
| 1959 | ABC020061659 | 2012 | 12 | + | + | + |
| 1960 | ABC020061788 | 2012 | 41 | + | - | - |
| 1961 | ABC020061794 | 2012 | 12 | + | - | + |
| 1962 | ABC020061807 | 2012 | 1 | + | - | + |
| 1963 | ABC020062000 | 2012 | 87 | + | - | + |
| 1964 | ABC020062022 | 2012 | 1 | - | + | + |
| 1965 | ABC020062090 | 2012 | 89 | + | - | - |
| 1966 | ABC020062096 | 2012 | 89 | + | - | - |
| 1967 | ABC020062119 | 2012 | 118 | + | - | - |
| 1968 | ABC020062551 | 2012 | 58 | + | - | + |
| 1969 | ABC020062597 | 2012 | 1 | + | - | + |
| 1970 | ABC020062598 | 2012 | 1 | + | - | + |
| 1971 | ABC020062612 | 2012 | 28 | + | - | - |
| 1972 | ABC020062635 | 2012 | 1 | + | - | + |
| 1973 | ABC020062639 | 2012 | 11 | - | + | + |
| 1974 | ABC020062649 | 2012 | 12 | + | + | + |
| 1975 | ABC020062808 | 2012 | 6 | + | - | + |
| 1976 | ABC020063091 | 2012 | 89 | + | - | - |
| 1977 | ABC020063121 | 2012 | 118 | + | - | - |
| 1978 | ABC020063308 | 2012 | 28 | - | + | - |
| 1979 | ABC020063495 | 2012 | 1 | + | - | + |
| 1980 | ABC020063685 | 2012 | 1 | - | + | + |
| 1981 | ABC020063694 | 2012 | 89 | + | - | - |
| 1982 | ABC020063827 | 2012 | 1 | - | + | + |
| 1983 | ABC020063836 | 2012 | 89 | + | + | + |
| 1984 | ABC020063839 | 2012 | 59 | - | + | + |
| 1985 | ABC020064113 | 2012 | 81 | + | - | + |
| 1986 | ABC020064176 | 2012 | 87 | + | - | - |
| 1987 | ABC020064178 | 2012 | 4 | + | - | + |
| 1988 | ABC020064338 | 2012 | 89 | + | - | - |
| 1989 | ABC020064339 | 2012 | 9 | + | - | + |
| 1990 | ABC020064456 | 2013 | 89 | + | - | - |
| 1991 | ABC020064522 | 2013 | 81 | + | - | + |
| 1992 | ABC020064525 | 2012 | 9 | + | - | - |
| 1993 | ABC020064535 | 2012 | 1 | + | - | + |
| 1994 | ABC020064756 | 2013 | 59 | + | - | + |
| 1995 | ABC020064783 | 2013 | 92 | + | - | + |
| 1996 | ABC020065404 | 2013 | 3 | + | - | - |
| 1997 | ABC020065505 | 2013 | 89 | - | + | - |
| 1998 | ABC020065506 | 2013 | 3 | - | + | - |
| 1999 | ABC020065508 | 2013 | 75 | + | + | + |
| 2000 | ABC020065512 | 2013 | 4 | + | - | + |

*^a^*These GAS isolates from patients with NF and/or STSS were collected in 2010-2013 by the CDC *Streptococcus Laboratory*; NF, necrotizing fasciitis; STSS, streptococcal toxic shock syndrome; SpeB secretion, lacking (-) or detected (+) in supernatant of overnight culture.
