# Supplementary material for: Contemporary Pharyngeal and Invasive emm1 and Invasive emm12 Group A Streptococcus Isolates Exhibit Similar In Vivo Selection for CovRS Mutants in Mice
Source: PLoS One. 2016 Sep 9;11(9):e0162742. doi: 10.1371/journal.pone.0162742 (PMC5017694; doi:10.1371/journal.pone.0162742)
Supplement: S2 Table — (DOCX) [file pone.0162742.s002.docx]

S2 Table. *emm* Genotype and SpeB Secretion of HMC Pharyngitis Isolates*^a^*

| Strain # | HMC # | *emm* genotype | SpeB Secretion |
| --- | --- | --- | --- |
| 2735 | HMC1 | *emm*75 | + |
| 2736 | HMC2 | *emm*89 | + |
| 2737 | HMC3 | *emm*12 | + |
| 2738 | HMC4 | *emm*1 | + |
| 2739 | HMC5 | *emm*89 | + |
| 2740 | HMC6 | *emm*3 | - |
| 2741 | HMC7 | *emm*12 | + |
| 2742 | HMC8 | *emm*12 | + |
| 2743 | HMC9 | *emm*1 | + |
| 2744 | HMC10 | *emm*3 | + |
| 2745 | HMC11 | *emm*1 | + |
| 2746 | HMC12 | *emm*87 | + |
| 2747 | HMC13 | *emm*12 | + |
| 2748 | HMC14 | *emm*9 | **-** |
| 2749 | HMC15 | *emm*1 | + |
| 2750 | HMC16 | *emm*12 | + |
| 2751 | HMC17 | *emm*12 | + |
| 2752 | HMC18 | *emm*3 | + |
| 2753 | HMC19 | *emm*4 | + |
| 2754 | HMC20 | *emm*238 | + |
| 2755 | HMC21 | *emm*89 | + |
| 2756 | HMC22 | *emm*1 | + |
| 2757 | HMC23 | *emm*1 | + |
| 2758 | HMC24 | *emm*89 | + |
| 2759 | HMC25 | *emm*28 | + |
| 2760 | HMC26 | *emm*22 | - |
| 2761 | HMC27 | *emm*111 | + |
| 2762 | HMC28 | *emm*77 | + |
| 2763 | HMC29 | *emm*85 | + |
| 2764 | HMC30 | *emm*9 | + |
| 2765 | HMC31 | *emm*48 | + |
| 2766 | HMC32 | *emm*2 | + |
| 2767 | HMC33 | *emm*12 | + |
| 2768 | HMC34 | *emm*89 | + |
| 2769 | HMC35 | *emm*6 | **-** |
| 2770 | HMC36 | *emm*28 | + |
| 2771 | HMC37 | *emm*28 | + |
| 2772 | HMC38 | *emm*1 | **-** |
| 2773 | HMC39 | *emm*82 | + |
| 2774 | HMC40 | *emm*12 | **-** |
| 2775 | HMC41 | *emm*1 | + |
| 2776 | HMC42 | *emm*81 | + |
| 2777 | HMC43 | *emm*2 | + |
| 2778 | HMC44 | *emm*2 | + |
| 2779 | HMC45 | *emm*1 | + |
| 2780 | HMC46 | *emm*87 | + |
| 2781 | HMC47 | *emm*28 | + |
| 2782 | HMC48 | *emm*87 | + |
| 2783 | HMC49 | *emm*58 | + |
| 2784 | HMC50 | *emm*59 | + |

*^a^*These pharyngeal isolates were collected in Seattle in 2014 by the Harborview Medical Center Clinical Microbiology Laboratory at University of Washington School of Medicine.
